# Identification and characterization of the long non-coding RNA NFIA-AS2 as a novel locus for body mass index in American Indians

**DOI:** 10.1038/s41366-023-01278-5

**Published:** 2023-02-17

**Authors:** Khushdeep Bandesh, Michael Traurig, Peng Chen, Wen-Chi Hsueh, Robert L. Hanson, Paolo Piaggi, Leslie J. Baier

**Affiliations:** Phoenix Epidemiology and Clinical Research Branch, National Institute of Diabetes and Digestive and Kidney Diseases, National Institutes of Health, Phoenix, AZ 85004 USA

**Keywords:** Genetics, Obesity

## Abstract

**Background:**

Genome-wide association studies have shown that body mass index (BMI), an estimate of obesity, is highly polygenic. Individual variants typically have small effect sizes, making it challenging to identify unique loci in under-represented ethnic groups which lack statistical power due to their small sample size. Yet obesity is a major health disparity and is particularly prevalent in southwestern American Indians. Here, we identify and characterize a new locus for BMI that was detected by analyzing moderate associations with BMI obtained in a population-based sample of southwestern American Indians together with the well-powered GIANT dataset.

**Methods:**

Genotypes for 10.5 million variants were tested for association with BMI in 5870 American Indians and 2600 variants that showed an association *P* < 10^−3^ in the American Indian sample were combined in a meta-analysis with the BMI data reported in GIANT (*N* = 240,608). The newly identified gene, *NFIA-AS2* was functionally characterized, and the impact of its lead associated variant rs1777538 was studied both in-silico and in-vitro.

**Results:**

Rs1777538 (T/C; C allele frequency = 0.16 in American Indians and 0.04 in GIANT, meta-analysis *P* = 5.0 × 10^−7^) exhibited a large effect in American Indians (1 kg/m^2^ decrease in BMI per copy of C allele). NFIA-AS2 was found to be a nuclear localized long non-coding RNA expressed in tissues pertinent to human obesity. Analysis of this variant in human brown preadipocytes showed that NFIA-AS2 transcripts carrying the C allele had increased RNA degradation compared to the T allele transcripts (half-lives = 9 h, 13 h respectively). During brown adipogenesis, *NFIA-AS2* featured a stage-specific regulation of nearby gene expression where rs1777538 demonstrated an allelic difference in regulation in the mature adipocytes (the strongest difference was observed for *L1TD1*, *P* = 0.007).

**Conclusion:**

Our findings support a role for *NFIA-AS2* in regulating pathways that impact BMI.

## Introduction

Obesity is a primary health concern that affects people from all ethnic backgrounds but there are considerable differences in its prevalence. A notable portion of this variation is due to the response of genes to an obesogenic environment which promotes weight gain during a high-calorie state [1–5]. Such genes can be identified by studying populations with a high prevalence of obesity, minimal genetic admixture, and less environmental variability. American Indians living in a southern Arizona community exhibit these features [6, 7], and many of these community members have participated in a longitudinal study of health in which height and weight were measured to calculate body mass index (BMI), a classical parameter to assess obesity. To identify obesity susceptibility genes in this community, our prior genetic studies have included replication of established BMI loci as well as genome-wide association studies to uncover new loci. However, a major challenge with identifying new loci for a trait in a relatively isolated population is that the sample size in a population isolate is limited; thus, genes with moderate effect sizes which influence polygenic diseases/traits will rarely achieve genome-wide significance. While well-established variants for BMI that show moderate associations are considered valid contributors in small samples, it is difficult to interpret the credibility of unreported variants that associate with BMI with comparably moderate statistical evidence. The focus of the current study is to identify loci for BMI in our American Indian sample that also have some effect in the well-powered Genetic Investigation of ANthropometric Traits (GIANT) consortium dataset. This strategy identified several known genes for BMI; however, among the genes never implicated with BMI was *NFIA-AS2*, a primate-specific long non-coding RNA (lncRNA).

## Methods

### Imputation and association analyses

Genotypes and BMI for American Indians were obtained from a community-based longitudinal study in Arizona from 1965 to 2007, where study exams included measurements of a 75‐g oral glucose tolerance test for type 2 diabetes (T2D) status defined by 1997 ADA criteria [8] and BMI. Since T2D is very common in this population and T2D progression and its treatment can affect BMI, the measure of BMI analyzed in the current study was the highest BMI recorded at any longitudinal examination at age ≥15 years when the volunteer was determined to be free from diabetes {*N* = 5870, mean age = 29.7 ± 11.4 years, mean BMI = 35.2 ± 8.4 kg/m^2^; among these *N* = 2571 were male, mean age = 29.1 ± 11.4 years, mean BMI = 33.9 ± 8.0 kg/m^2^; and *N* = 3299 were female, mean age = 30.2 ± 11.5 years, mean BMI = 36.1 ± 8.5 kg/m^2^}. The study was approved by the Institutional review board of the National Institute of Diabetes and Digestive and Kidney Diseases (National Institutes of Health) and all participants provided written informed consent.

Study volunteers were previously genotyped using a custom genotyping array (Affymetrix) designed specifically for this population based on whole-genome sequence data. Variants on this array that passed all quality control metrics (*N* = 515,723) captured 91% of all common variants with minor allele frequency (MAF) ≥ 0.05 and 56% of low-frequency variants (MAF = 0.01 − 0.05) across the genome [4, 9]. These 515,723 single nucleotide polymorphisms (SNPs) were used as a scaffold for imputation using the reference panel of 296 American Indians which were genotyped by whole-genome sequencing. For the reference panel, sequencing reads were aligned to the Human genome build Hg38 using bwa; [10] genotype calling was made using two walkers HaplotypeCaller and GenotypeGVCF of GATK version 3.5 [11]. For imputation, we removed the genomic variants with more than 2 alleles from the reference panel. We adopted the best practices for pre-phasing and imputation as suggested in the IMPUTE2 website (https://mathgen.stats.ox.ac.uk/impute/impute_v2.html). In brief, we phased our genotype data by estimating haplotypes by SHAPEIT [12] using the duoHMM method which incorporates pedigree information in our data for a more accurate estimate of the haplotypes. The genotypes with Mendelian errors were set to missing, which could be imputed back together with the missing calls, using other samples as reference. The phased data was divided into 5 Mb segments and the missing genotypes in the reference panel were typed with IMPUTE2 executed using default parameters [13]. The imputed allele dosage data was transformed to the long text format understood by SAS version 9 [14]. Imputed variants with MAF ≥ 0.05 and imputation INFO score ≥ 0.5 were analyzed for association with rank normalized BMI values using normalizing inverse Gaussian transformation. The association between BMI and genotype dosages (assuming additive model) was adjusted for age, sex, birth year, first five principal components, and was analyzed using linear “mixed” model that accounted for genetic relationships among individuals (estimated by determining identity by descent (IBD) across genotyped markers among all pairs of individuals) as previously described [4]. Variants that showed association with BMI in American Indians at a *P*-value < 0.001 were considered for meta-analysis with the BMI results in the GIANT consortium database [15]. GIANT BMI association data was downloaded from the consortium portal (https://portals.broadinstitute.org/collaboration/giant/index.php/GIANT_consortium_data_files). In total, 2600 variants that were present in both the American Indian and GIANT datasets were meta-analyzed by combining *P*-values using Stouffer’s method. P-values for heterogeneity were also determined based on Cochran’s Q statistic, calculated from the effect sizes and their standard errors.

### Tissue expression

Total RNA of human pituitary, adrenal and adipose, and cDNAs of brain, hypothalamus, skeletal muscle, liver, kidney, and pancreas were purchased from Takara Bio (San Jose, CA, USA). RNA was converted to cDNA using ProtoScript II First Strand cDNA Synthesis Kit (New England Biolabs Inc., Ipswich, MA, USA). Primers specific for *NFIA-AS2* transcript variant 1 were used for amplification. PCR products were sequenced for confirmation.

### Cellular localization

RNA from nuclear and cytoplasmic fractions of human brown preadipocytes was purified using a commercial extraction kit (Norgen Biotek Corp., ON, Canada) and NFIA-AS2 presence was screened by RT-PCR. Different RNA markers were analyzed to check any cross-contamination between the fractions: 5S rRNA (nuclear), β-actin (cytoplasmic), and 28S rRNA (both nuclear and cytoplasmic). No amplification was seen in the RT (-) samples. Assay was repeated on 4 independent days.

### RNA decay

Expression plasmids for *NFIA-AS2* rs1777538 T and C allele-specific transcripts and eGFP were purchased from GenScript (Piscataway, NJ, USA). Each *NFIA-AS2* plasmid (1000 ng) was co-transfected with eGFP (1000 ng) (to monitor transfection efficiency) in PAZ6 cells using Lipofectamine 3000 (Invitrogen, Waltham, MA, USA). After 24 h, global transcription was inhibited using 15 μg of actinomycin D (Sigma-Aldrich, St. Louis, MO, USA) per ml of complete growth medium. Cells were harvested before actinomycin D treatment (time 0) and at 3-, 6-, and 24-hours post-treatment, and *NFIA-AS2* and *TBP* expression were measured by qRT-PCR on a QuantStudio 12K-Flex Real-Time PCR system (Applied Biosystems, Waltham, MA, USA). The percentage of remaining transcripts was calculated at each time-point by 2^-ΔCt^ method and transcript half-lives (T_1/2_) were computed from rate of decay using the expression $\frac{{{{{\mathrm{ln}}}}(2)}}{{ - (slope\;of\;the\;graph)}}$. Assays were done in triplicate and performed on 3 separate days.

### MicroRNA mimic assays

We used the lncRNASNP2 database [16] to determine whether rs1777538 disrupts a microRNA binding site. To measure endogenous expression levels of miR4270, miR6754-5p, and miR4441 in HEK293 cells (ATCC, Manassas, VA, USA) and brown preadipocytes (ABM, BC, Canada), we isolated total RNA including miRNAs using miRNeasy Kit (Qiagen, Hilden, Germany) which was reverse-transcribed using TaqMan Advanced miRNA cDNA Synthesis Kit (Applied Biosystems) and miRNA expression was measured by qRT-PCR using TaqMan Advanced miRNA assays and TaqMan Fast Advanced Master-Mix (Applied Biosystems). For binding assays, Mission miRNA mimics (50 nM) for miR4270, mir6754-5p (Sigma-Aldrich, St. Louis, MO, USA) were co-transfected with *NFIA-AS2* rs1777538 T or C allele-specific expression vector (500 ng) and eGFP (500 ng) in HEK293 cells cultured in EMEM (ATCC) supplemented with 10% FBS (Gibco, Thermo Fisher Scientific). Transfections were performed in an antibiotic-free medium, and 24 h post-transfection HEK293 cells were collected, and *NFIA-AS2* rs1777538 T and C allele-specific gene expression were measured by qRT-PCR using a TaqMan expression assay (Thermo Fisher Scientific). To further examine the effects of rs1777538 on miR4270 and mir6754-5p binding, 50 bp fragments containing the putative miRNA target site with either the T or C allele were cloned into the psiCHECK-2 miRNA target expression vector (Promega, Madison, WI, USA) at the 3’ end of the Renilla luciferase gene. The constructs were co-transfected with either miR4270 or miR6754-5p miRNA mimics (Sigma-Aldrich) or with Mission miRNA, negative control 1 (Sigma-Aldrich) into HEK293 cells using lipofectamine 3000 (Thermo Fisher Scientific). Forty-eight hours post-transfection, the cells were analyzed for Renilla luciferase activity and normalized with firefly luciferase activity using the Dual-Luciferase Reporter Assay System (Promega). Transfections were done in triplicate and luciferase assays were performed 3 time on different days.

### Measuring gene expression during differentiation

PAZ6 immortalized human brown preadipocytes were purchased from Applied Biological Materials Inc. (ABM) and cultured in Prigrow-IV medium (ABM) supplemented with 10% FBS (Gibco), 1% L-glutamine (ABM), 15 mM HEPES (Sigma-Aldrich), and 1% Penicillin/Streptomycin (ABM). For differentiation, 1.5 ×10^5^ cells were seeded per well in 12-well plates pre-coated with extracellular matrix (ABM) and were grown to 100% confluency. After additional 48 h post-confluency, differentiation was induced (Day 0) by replacing the growth medium with a medium consisting of Prigrow-IV supplemented with 5% FBS (Gibco), 1% Penicillin/Streptomycin (ABM), 15 mM HEPES (Sigma-Aldrich), 33 μM biotin (Sigma-Aldrich), 17 μM calcium pantothenate (Sigma-Aldrich), 1 nM triiodothyronine (Sigma-Aldrich), 100 nM dexamethasone (Sigma-Aldrich), 1 μM pioglitazone (Sigma-Aldrich), 500 nM human insulin (Sigma-Aldrich) and 0.25 mM 3-Isobutyl-1-methylxanthine (IBMX) (Sigma-Aldrich). On Day 4, differentiation medium was replaced with a medium containing all supplements except IBMX, thereafter the medium was refreshed every two days for next ten days. On Day 14, mature adipocytes were observed. Adipocyte development was tracked by Oil-O-Red staining (Abcam) and expression of various marker genes - *EBF2*, (brown adipose lineage); *UCP1, PGC1A, CITED1* (thermogenesis); *PPARG, FABP4, ADIPOQ, LEP* (adipogenesis); *PANK1, ELOVL3* (lipid metabolism); and *GLUT4* (glucose hemostasis). To rule out the possibility of the development of beige adipocytes, the expression of the classic cell-type specific genes was analyzed – *ASC1* (white fat); *P2XR5* (brown fat); and *PRDM16* (beige fat) [17]. Only *P2XR5* expression was detected in PAZ6 preadipocytes (average Ct = 24.9); its high expression persisted during differentiation (average Ct = 25.5, mature brown adipocytes). Genes *ASC1* and *PRDM16* did not express in PAZ6 preadipocytes (Ct = undetermined) and showed very weak gene expression upon differentiation (average Ct = 33.3, *ASC1*; 32.2, *PRDM16*). Cells were harvested at the indicated time-points and the neighboring gene expression was quantified by qRT-PCR using PowerUp SYBR-Green master mix (Applied Biosystems). *NFIA-AS2* expression was measured using TaqMan gene expression probes. Real-time assays were done in duplicate, and the expression of each gene was normalized with *TBP*. Differentiation was repeated on three independent days done in triplicate.

### NFIA-AS2 overexpression

Uninduced PAZ6 brown preadipocytes at Day zero (D0) and differentiating preadipocytes at D4, D6, and D14 were co-transfected with 1000 ng of either the *NFIA-AS2* T or C allele expression plasmids and 1000 ng of eGFP plasmid. Differentiation and gene expression analysis at D0, D4, D6, and D14 was conducted as described in the previous section. Expression of neighboring genes was normalized with *LMNB1*. Overexpression experiments were conducted on 3 separate days in triplicates per experiment.

## Results

### Genome-wide association analysis

**Fig. 1:**
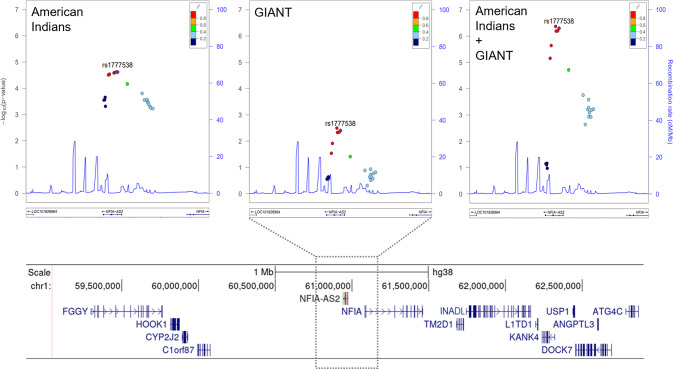
Regional BMI association results for the *NFIA-AS2* locus in American Indians show stronger associations after meta-analysis with the GIANT data. Rs1777538 (shown as purple diamond) presented stronger association following meta-analysis. SNP coloring is based on linkage disequilibrium (LD) with rs1777538 in 1000 Genomes AMR population. Genetic recombination rates (cM/Mb) are shown as blue spikes. The lower panel displays the genomic segment presented in the regional association plot and the flanking region showing 13 protein-coding genes that exist in *NFIA-AS2* neighborhood, UCSC Genome Browser build 38 [44].

Association analysis of approximately 10.5 million imputed variants in 5870 American Indians identified 7733 variants that associated with BMI with a *P* < 0.001. A threshold of *P* < 10^−3^ was selected for further study because this is the level of significance obtained for many well-established BMI genes (e.g., variants in *FTO*) in this sample. Among these 7733 variants with moderate evidence for association with BMI in American Indians, 2600 were also present in the GIANT dataset from multiple ancestries (*N* = 240,608) (Supplementary Fig. 1). A meta-analysis of the American Indian and GIANT BMI association data for these 2600 variants was conducted (Supplementary Table 1) and the lead variants for the top genes are shown in Table 1. Among top signals, six of the genes (*FTO, TMEM18, PRKD1, GRP, NLRC3*, and *MC4R*) are well-established GWAS loci for BMI [9], while another gene (*MAP2K3*) has been previously reported as a BMI locus in American Indians which replicates in GIANT [18]. Furthermore, the gene *RORA* has been implicated in severe obesity in humans and mice [19]. Among the remaining signals, the T2D knowledge-portal provides strong SNP replication data for rs17522122 in *AKAP6* (*P* = 10^−23^) and rs9823593 in *LSAMP* (*P* = 10^−10^), and some support for rs4950106 in *MIR137HG* (*P* = 10^−5^). Thus, among our top genes for BMI, the only truly novel locus is *NFIA-AS2*, and it was prioritized for follow-up. The genomic region flanking the lead *NFIA-AS2* variant rs1777538 showed significant improvement in association with BMI after meta-analysis (Fig. 1). Genotypes for rs1777538 were verified by direct genotyping in 5479 American Indian samples using a TaqMan genotyping assay (Applied Biosystems). The concordance for imputed genotyping versus direct genotyping was 99.7%. The minor allele C of rs1777538 is more common in the American Indian as compared to the GIANT datasets (frequency = 0.16 vs 0.04) and each copy of the C allele resulted in an average BMI decrease of 0.117 SD (about 1 kg/m^2^) in American Indians, as compared with 0.022 SD in Europeans.

**Table 1:** Top independent signals associated with BMI identified by the American Indian + GIANT meta-analysis.

			American Indians (*N* = 5870)			GIANT (*N* = 167,425–239,749)			Meta-analysis	
Variant	Loci	P/R^a^	Freq_P_	Effect_P_	*P*	Freq_P_	Effect_P_	*P*	*P*_het_	*P*
rs1421085	*FTO*	T/C	0.85	−0.100	4.6 × 10^−4^	0.55	−0.075	2.0 × 10^−107^	0.39	1.1 × 10^−73^
rs2867110	*TMEM18*	C/G	0.15	−0.126	1.0 × 10^−5^	0.12	−0.060	7.5 × 10^−46^	0.02	3.3 × 10^−40^
rs1569980	*PRKD1*	T/C	0.26	−0.082	4.2 × 10^−4^	0.21	−0.021	9.9 × 10^−7^	0.009	2.8 × 10^−9^
rs17522122	*AKAP6*	G/T	0.61	−0.075	3.8 × 10^−4^	0.53	−0.016	3.9 × 10^−6^	0.006	7.6 × 10^−9^
rs1062557	*GRP*	C/A	0.69	−0.085	1.7 × 10^−4^	0.20	−0.017	1.1 × 10^−4^	0.003	7.8 × 10^−8^
rs16942379	*MAP2K3*	T/C	0.32	−0.075	7.2 × 10^−4^	0.48	−0.016	3.1 × 10^−5^	0.009	9.7 × 10^−8^
rs4950106	*MIR137HG*	G/A	0.17	−0.102	2.2 × 10^−4^	0.63	−0.012	2.7 × 10^−4^	0.001	2.2 × 10^−7^
rs12445430	*NLRC3*	C/T	0.82	−0.091	6.1 × 10^−4^	0.81	−0.017	2.0 × 10^−4^	0.006	3.7 × 10^−7^
**rs1777538**	***NFIA-AS2***	**C/T**	**0.16**	**−0.117**	**2.4** **×** **10**^**−5**^	**0.04**	**−0.022**	**4.0** **×** **10**^**−3**^	**0.0009**	**5.0** **×** **10**^**−7**^
rs9823593	*LSAMP*	G/C	0.09	−0.119	6.3 × 10^−4^	0.22	−0.017	3.7 × 10^−4^	0.004	8.3 × 10^−7^
rs16943429	*RORA*	C/T	0.32	−0.093	2.9 × 10^−5^	0.20	−0.012	8.5 × 10^−3^	0.0004	1.3 × 10^−6^
rs17066829	*MC4R*	T/A	0.45	−0.073	6.3 × 10^−4^	0.59	−0.012	6.6 × 10^−4^	0.005	1.4 × 10^−6^

*P* values for the American Indian BMI associations were adjusted for age, sex, birth-year, the first five genetic principal components and identity by descent. Meta-analysis was performed using Stouffer’s method based on combining *P* values.

*Freq*_*P*_ frequency of the protective allele, *Effect*_*P*_ effect size for the protective allele, *P*_*het*_ the posterior probability of heterogeneity.

^a^*P* protective allele for obesity, *R* risk allele for obesity.

### NFIA-AS2 is expressed in tissues relevant to obesity

*NFIA-AS2*, an uncharacterized primate-specific lncRNA, maps antisense to the long protein-coding transcript of *NFIA*, within intron 1. *NFIA-AS2* has 3 transcripts, variant 1 (NR_110617.1, *NFIA-AS2* v1) and variant 2 (NR_110618.1) and variant 3 (ENST00000665665.1; Supplementary Fig. 2). Rs1777538 (T/C) detected in our meta-analysis, is located in exon 2 of *NFIA-AS2* v1, hence only the v1 transcript isoform of *NFIA-AS2* was investigated in our study.

**Fig. 2:**
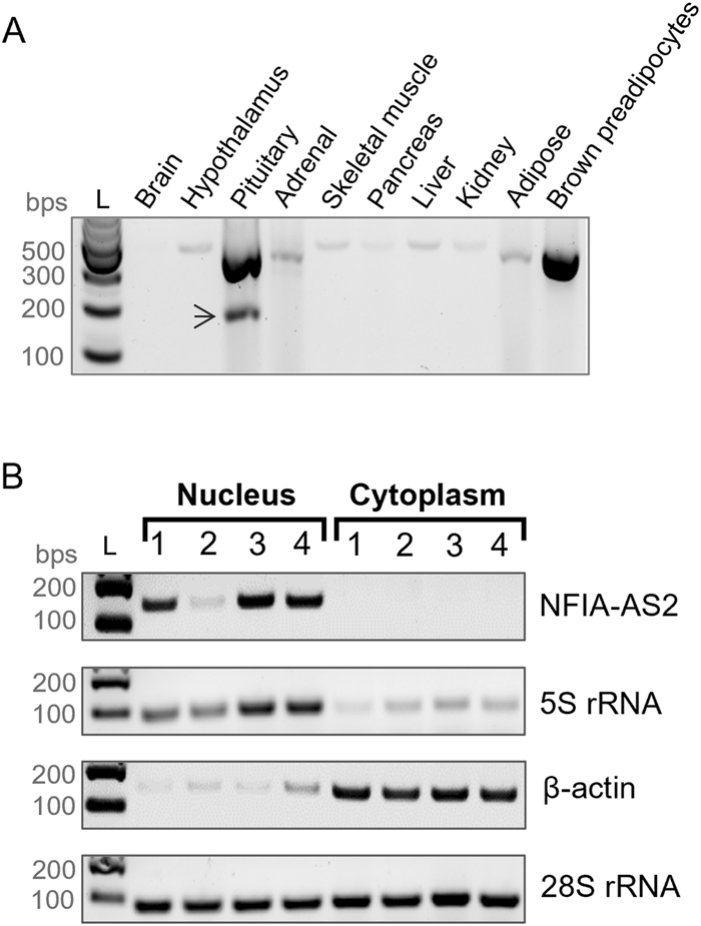
NFIA-AS2 is a nuclear lncRNA expressed in tissues involved in human obesity. **A** RT-PCR gel shows *NFIA-AS2* is expressed in various human tissues that play a role in the pathogenesis of obesity and obesity-related traits. The arrow is pointing to a novel alternative *NFIA-AS2* transcript identified in the pituitary which is missing exons 3 and 4. **B** Results for the nuclear and cytoplasmic fractionation of PAZ6 brown preadipocytes show that *NFIA-AS2* transcripts are localized to the nucleus. Genes of interest were PCR amplified from the isolated nuclear and cytoplasmic RNA fractions and PCR products were run on 1.5% agarose gel. No amplification was seen in the RT (-) samples. Nuclear marker: 5S rRNA; cytoplasmic marker: β-actin; nuclear and cytoplasmic marker: 28S rRNA. The experiment was repeated 4 times on separate days. L, 100 bp DNA ladder.

*NFIA-AS2* is expressed in various human tissues that play a role in the development of obesity (Fig. 2A). These tissue cDNAs were from commercial source and not related to American Indian ancestry. In particular, *NFIA-AS2* has high levels of expression in brown preadipocytes. Unexpectedly, while examining *NFIA-AS2* tissue expression we detected a novel alternative transcript isoform that is highly expressed in human pituitary and lacks exons 3 and 4. Furthermore, we also examined the expression of the genes flanking *NFIA-AS2* (Fig. 1, *N* = 13) in various tissues in mouse and observed the highest neighboring gene expression in the brown adipose tissue characterized by an abundance of polyA+ RNA sequencing reads (ENCODE consortium, Supplementary Fig. 3). Together, our data supported brown preadipocytes as a biologically relevant cell line for our assays.

### NFIA-AS2 is localized in the nucleus

We investigated the cellular localization of *NFIA-AS2* lncRNA in PAZ6 human brown preadipocytes and NFIA-AS2 was found in the nuclear fraction (Fig. 2B) suggesting that it could potentially be involved in regulating gene expression.

### Rs1777538 variant affects NFIA-AS2 RNA degradation

**Fig. 3:**
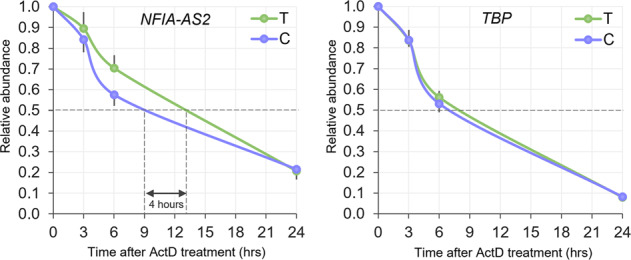
RNA degradation kinetics for *NFIA-AS2* harboring either the rs1777538 T or C allele. Global transcription in brown preadipocytes was blocked with actinomycin D (ActD) at a 0-hour time point. Cells were collected at 3, 6, and 24 h post-ActD treatment for RNA isolation and subsequent measurement of *NFIA-AS2* transcript levels. *TBP* was used as a control. Transcript levels for the ActD treated cells are shown relative to untreated cells (0 h). Results are from 3 independent experiments each done in triplicate and shown as mean+/− SE.

The effect of rs1777538 on *NFIA-AS2* RNA structure and stability was evaluated. *In-silico* analysis of *NFIA-AS2* RNA secondary structure did not identify a significant difference in minimum free energy (MFE) for RNA harboring either the T or C allele at rs1777538 (MFE = −356.9 kcal/mol and −357.8 kcal/mol respectively, Supplementary Fig. 4) [20]. To assess an impact on stability, we analyzed the rate of decay of NFIA-AS2 following transcription inhibition with actinomycin D and observed a significant difference of 4 h in the half-lives between the *NFIA-AS2* T and C allele RNA transcripts, where the C allele transcripts had increased degradation compared to the T allele transcripts (T_1/2(C)_ = 9 h, T_1/2(T)_ = 13 h, Fig. 3) and there was no significant difference in *TBP* mRNA half-life (Fig. 3). These results suggest that rs1777538 alters *NFIA-AS2* RNA turnover and potentially influences its downstream function.

### Rs1777538 variant is predicted to disrupt a microRNA binding site

**Fig. 4:**
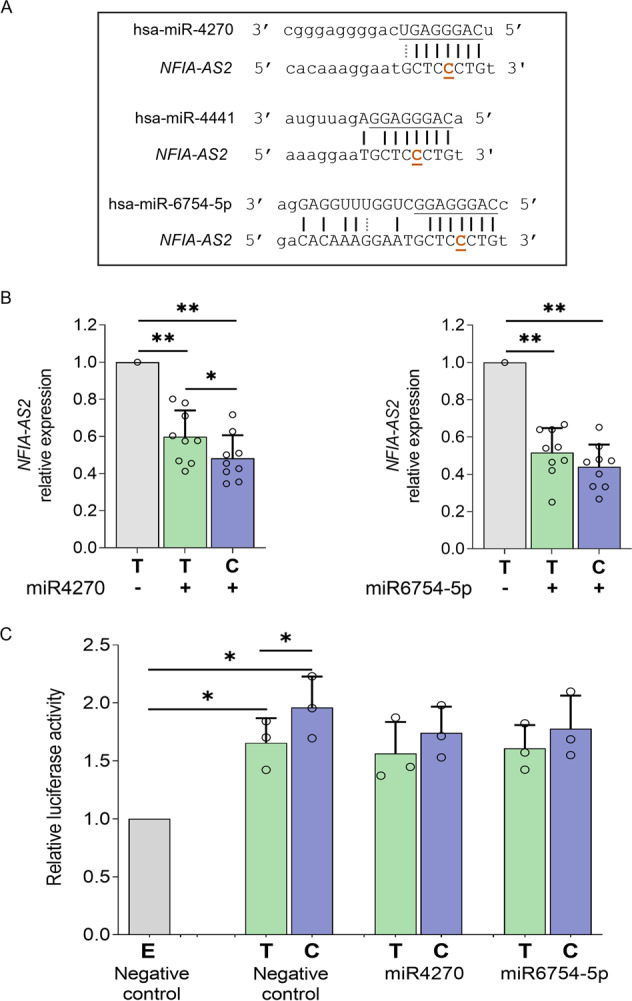
The rs1777538 T > C variant destabilizes NFIA-AS2 affecting a miRNA target site. **A** Rs1777538 T > C variant disrupts a miRNA target site. The rs1777538 variant is located within the seed region (underlined) for a putative target site for 3 miRNAs miR4270, miR6754-5p, and miR4441 [figure adapted from lncRNASNP2 database (16)]. The rs1777538 C allele is shown in orange font and underlined. **B** Both miRNA mimics miR4270 and miR6754 significantly decrease *NFIA-AS2* levels in HEK293 cells. Data was normalized with eGFP (transfection efficiency) and *TBP*. **C** Relative luciferase activity for the binding of miR4270 and miR6754-5p to *NFIA-AS2* in an allele-specific manner. The miRNA target site constructs carrying rs1777538 alleles (C) or (T) were cotransfected with either a human miRNA mimic or a negative control in HEK293 cells. Observed luciferase activity was normalized with respect to the empty vector (E). Results are from 3 independent experiments each done in triplicate and shown as mean + SD. Data points per group are shown as hollow circles. Statistical analysis was carried out by Student’s *t* test. *P < 0.05; **P ≤ 10^−5^.

One mechanism whereby a variant could affect RNA decay is via an effect on miRNA binding (miRNA target loss or gain). We found that rs1777538 is located within the seed region for a putative target site for 3 miRNAs miR4270, miR6754-5p, and miR4441 (Fig. 4A), and the rs1777538 T allele is predicted to alter the miRNA binding site (miRNA target loss). This predicted miRNA binding site was not found anywhere else in the *NFIA-AS2* RNA sequence (data not shown). These results may underlie the increase in RNA degradation for the *NFIA-AS2* transcript containing the C allele (unaltered miRNA binding site) compared to the T allele transcripts (altered target site). This prediction was experimentally examined in HEK293 cells which have a very low endogenous expression of *NFIA-AS2* (data not shown). Also, expression levels for miR4270 and miR6754-5p were very low (average Ct = undetermined and 35.7, respectively) while miR4441 was highly expressed (average Ct = 19.1). To avoid spurious results due to high expression of endogenous miR4441, only miR4270 and miR6754-5p miRNA were investigated. *NFIA-AS2* expression plasmids carrying either the rs1777538 T or C allele were co-transfected with miR4270 and miR6754-5p miRNA mimics in HEK293 cells. We found that both miRNAs target *NFIA-AS2* and significantly decreased expression levels compared to the control (no miRNA) (Fig. 4B). There was also a modest difference between the rs1777538 T and C alleles with miR4270 (*P* = 0.04) presenting a higher impact on the transcripts carrying the C allele. To further investigate the effects of rs177538 on miR4270 and mir6754-5p binding efficiency, 50 bp fragments containing the predicted miRNA binding site with either the T or C allele were cloned into a miRNA target reporter vector. In vitro analysis using these reporter constructs co-transfected with either miR4270 or miR6754-5p miRNA mimics showed that the cloned 50 bp region can function as a transcriptional regulatory element as indicated by an approximately 2-fold increase in luciferase activity compared to the empty reporter vector (Fig. 4C). There also appears to be a modest difference in luciferase activity between the rs1777538 T and C alleles. The two miRNAs do not have a significant effect on the expression of the luciferase gene containing the predicted miRNA binding site (Fig. 4C). This could be due to the confounding effects of the increased expression levels due to the putative *cis*-regulatory element located in the 50 bp fragment.

### NFIA-AS2 is not a miRNA sponge for its neighbors

LncRNAs that bind miRNAs can upregulate gene expression by preventing the interaction of bound miRNA with its target mRNAs, thus serving as a miRNA sponge. NFIA-AS2 is reported to sponge miR-665-3p [21]. Furthermore, analysis of the sequence around rs1777538 in the ENCODE Regulation database supports that this variant is positioned in a genomic region highly enriched with the histone modifications H3K27Ac and H3K4Me1, a signature characteristic of active enhancers (Supplementary Fig. 2). Given that, we studied the ‘miRNA sponging’ ability of NFIA-AS2 in *cis* for 13 genes that reside in a 3 Mb region flanking *NFIA-AS2* (Fig. 1). We inspected the changes in their endogenous gene expression in the presence of miR4270 and miR6754-5p in HEK293 cells. None of the investigated genes were targeted by miR4270 and miR6754-5p, reducing the likelihood that ‘miRNA sponging’ is an important mechanism for NFIA-AS2 activity (Supplementary Fig. 5).

### NFIA-AS2 affects neighboring gene expression in brown adipocytes

**Fig. 5:**
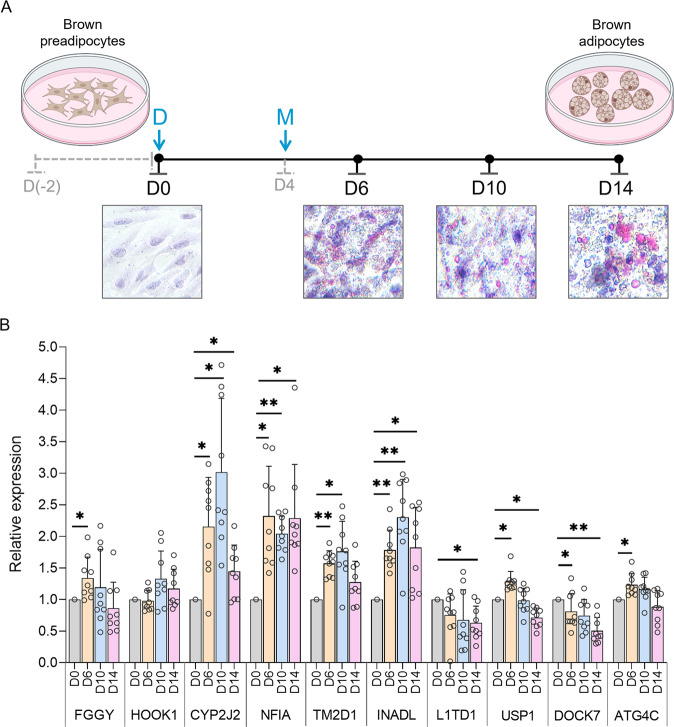
Gene expression analysis for the 13 *NFIA-AS2* neighboring genes during brown preadipocyte differentiation. **A** Schematic showing PAZ6 brown adipogenesis over a period of 14 days assessed by Oil-O-Red staining at days D0 (brown preadipocytes), D6, D10, and D14 (brown adipocytes). D: induction of differentiation; M: initiation of maturation. **B** Expression patterns for the *NFIA-AS2* neighboring genes during brown adipogenesis. Gene expression was normalized with *TBP*. Preadipocyte differentiations were repeated 3 times on separate days and each differentiation had 3 replicates. Data is shown as mean + SD. Data points per group are shown as hollow circles. Significance was calculated with respect to D0 using the Student’s *t* test. *P ≤ 10^−3^; **P ≤ 10^−5^.

We examined the expression pattern of 13 neighboring genes during brown adipogenesis. Human brown preadipocytes were differentiated over 14 days (Fig. 5A) and brown adipogenesis was verified by analyzing the expression of marker genes (Supplementary Fig. 6). Investigation at 4 different Days (D)/stages: D0 (preadipocytes), D6 (differentiating preadipocytes), D10 (immature adipocytes), and D14 (terminally differentiated adipocytes) indicated differential expression of all genes during adipogenesis except for *C1orf87*, *KANK4*, and *ANGPTL3*, which were not expressed at any stage in PAZ6 cells (Fig. 5B). Immediate neighbors of *NFIA-AS2*, *CYP2J2*, *NFIA*, *TM2D1*, and *INADL* were strongly upregulated upon differentiation and showed the highest expression at D10, except *NFIA* which had slightly lower expression than the D6 and D14. Genes distant to *NFIA-AS2*, *L1TD1* and *DOCK7* were downregulated in mature brown adipocytes compared to preadipocytes; while *FGGY*, *USP1* and *ATG4C* were modestly upregulated upon induction of differentiation but downregulated in mature adipocytes (Fig. 5B). *NFIA-AS2* gene expression did not change during differentiation (data not shown).

**Fig. 6:**
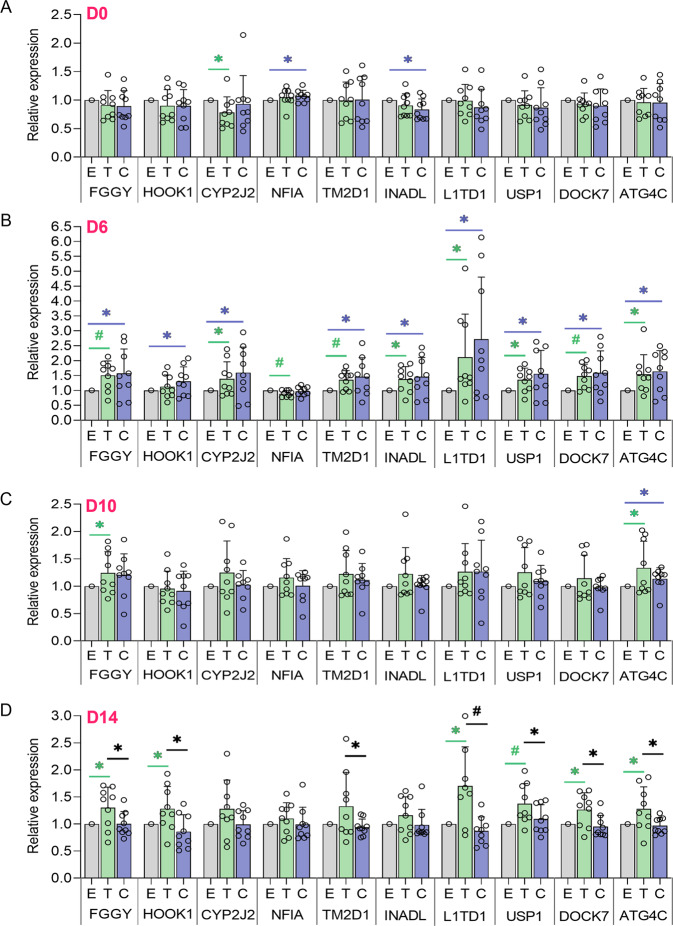
Effects of *NFIA-AS2* rs1777538 variant on neighboring gene expression during brown adipogenesis. Uninduced brown preadipocytes (D0) and differentiating preadipocytes at D4, D6, and D14 were transfected with either the *NFIA-AS2* T or C allele expression plasmids. Expression levels for the nearby genes were measured 24 h post-transfection for D0, D6, D10, and D14 and are shown relative to the empty vector control (E). All gene expression levels were normalized with *LMNB1*. The differentiations and transfections were performed 3 times on separate days and done in triplicate per experiment. Data is shown as means + SD. Data points per group are shown as hollow circles. Significance was calculated using the Student’s *t* test. *P = 0.01–0.05; ^#^*P* = 0.003–0.009.

To investigate the effects of rs1777538, T and C allele-specific *NFIA-AS2* transcripts were transiently overexpressed at days D0, D6, D10, and D14. We did not observe any considerable changes in neighboring gene expression at the preadipocyte stage (D0) (Fig. 6A). Once the cells were shifted to the maturation phase (D6), overexpression of both *NFIA-AS2* T and C allele transcripts induced expression for all genes except for *NFIA* which showed some reduction in expression with the T allele (Fig. 6B). Meanwhile, *NFIA-AS2* C allele transcripts resulted in a greater magnitude of upregulation in nearby gene expression compared to the T allele transcripts, however, the differences did not reach statistical significance. (Fig. 6B). At D10, there was an observable decline in both the upregulation and differential expression for the neighboring genes and this was especially evident for *L1TD1* (Fig. 6C). However, in mature brown adipocytes (D14) the variable effects of the *NFIA-AS2* allele-specific transcripts on gene expression were opposite compared to D6. Contrary to D6, at D14, overexpression of the *NFIA-AS2* T allele transcript led to an increase in gene expression for many neighboring genes compared to the C allele transcript (strongest allelic difference observed for *L1TD1*) (Fig. 6D). These results indicate that the effects on gene expression by *NFIA-AS2* may be stage-specific (temporal) and that *NFIA-AS2* may have different functional roles for the D6 (immature adipocytes) and D14 (mature adipocytes) stages to upregulate gene expression.

## Discussion

Our meta-analysis of genetic variants that nominally associate with BMI in both American Indians and individuals from the GIANT consortium identified several loci that have been extensively studied for their role in BMI and several others that have been statistically associated with BMI but warrant a biologic follow-up for a potential role in body weight. The current report is focused on follow-up of one of these loci, *NFIA-AS2*, since this locus had never been linked to BMI yet it was among the strongest signals in the American Indian data. *NFIA-AS2*’s namesake *NFIA* has been linked to brown adipocyte differentiation [22, 23] and prior studies have suggested involvement of other lncRNAs in obesity [24–28]; however, *NFIA-AS2* has never before been reported to affect body weight.

Our functional studies suggest that the BMI-associated variant rs1777538 may affect *NFIA-AS2* RNA decay (turnover rate) which in turn may alter cellular function. In addition, we show that the variant is in a *cis*-regulatory element (CRE) and may alter a transcription factor binding site where the T allele results in lower gene expression. The putative CRE is predicted to interact with the promoters of long isoform of *NFIA* (ENST00000371191.5) and *NFIA-AS2* (Supplementary Fig. 2); and the rs1777538 variant is reported to be an eQTL for *NFIA-AS2* in tibial nerve tissue where the TT genotype is also associated with lower gene expression (Supplementary Fig. 7). Despite a greater number, C-allele transcripts showed higher RNA degradation compared to the transcripts with the T allele. This increased decay of *NFIA-AS2* RNAs with C allele could be in part due to stronger binding of miRNAs to their predicted target which encompasses the C/T variant at rs1777538, e.g., miR4441 which is highly expressed in brown preadipocytes (average Ct= 19.8). However, because of the small difference in binding efficiency for miR4270 between the T and C alleles of *NFIA-AS2* transcripts, we do not think that differential miRNA binding is the sole reason for increased RNA decay. Of the obesity-related tissues we examined, *NFIA-AS2* is highly expressed in brown preadipocytes. LncRNAs play an important role in brown adipose metabolism (e.g., adipogenesis, non-shivering thermogenesis) including Blnc1, H19, and lnc-dPrdm16 among others [28] and they can function in *cis* (locally) by regulating the spatiotemporal expression of neighboring protein-coding target genes [24, 29]. We demonstrated that overexpression of *NFIA-AS2* T and C allele-specific transcripts during brown adipogenesis influenced the expression of nearby genes in a temporal manner at D6 and D14. Moreover, we also observed differential effects on gene expression between the *NFIA-AS2* T and C allele transcripts particularly in mature brown adipocytes (D14).

The differential regulation of *DOCK7* at D14 is intriguing. A previous study has shown that *Misty* mice which harbor a premature stop codon in the *Dock7* gene lack brown fat pads [30]; and, in the second pair of later studies, it was also shown that *Misty* mice have reduced brown adipose innervation and impaired function (thermogenesis) [31, 32]. Among others, *CYP2J2* expression has also been linked to brown adipogenesis [33], while *ATG4C* belongs to a family that includes similar proteins involved in brown adipocyte metabolism [34]; we noticed a constant variation in *ATG4C* expression whenever *NFIA-AS2* was overexpressed during differentiation. The connection between the gene, *L1TD1* which showed the largest differential regulation in mature adipocytes, and brown adipose metabolism is unclear. L1TD1 functions as an RNA-binding protein in various cellular processes including RNA degradation, splicing, translation, and protein trafficking [35, 36]. Rs1777538 C-allele which predicted leanness in our association studies and caused higher *NFIA-AS2* degradation, demonstrated significant reduction in *L1TD1* expression in brown adipocytes (D14). We speculate that L1TD1 might be involved in the decay of *NFIA-AS2* transcripts. The only report, to date, linking *L1TD1* and obesity is a study that examined genome-wide DNA methylation patterns in preadipocytes isolated from lean individuals and individuals with obesity and T2D, they observed differential methylation at *L1TD1* promoter (↑ methylation in lean group) and a 2-fold increase in *L1TD1* expression for the group with obesity and T2D [36]. Considering the functional effects of *NFIA-AS2* on multiple genes in the neighborhood, we suspect that the overexpression of the affected genes influences the entire differentiation, and the response may not be necessarily mediated by the direct action of *NFIA-AS2* lncRNA.

In addition to human brown preadipocytes, *NFIA-AS2* is also highly expressed in the pituitary and moderately expressed in the hypothalamus and adrenal gland (Fig. 2A). These 3 tissues form the hypothalamic-pituitary-adrenal (HPA) axis which assumes a central role in the neuroendocrine control of obesity. HPA axis controls the stress response in the body by regulating the production and secretion of neurohormones that influence energy intake and body weight [37]. In general, a higher BMI is associated with a larger volume of pituitary gland [38] and a hyperactivity of HPA axis [39]. Given the high expression of *NFIA-AS2* in HPA tissues and strong association with BMI, future studies can explore the biological role of *NFIA-AS2* in HPA activity.

In conclusion, we identified a lncRNA, *NFIA-AS2*, as a novel BMI locus using a multiethnic meta-analysis that included American Indians from southern Arizona and individuals from the GIANT consortium. Functional characterization of lead *NFIA-AS2* signal rs1777538 showed that its minor allele (C) promotes *NFIA-AS2* RNA decay and downregulates several neighboring genes during later stages of brown adipogenesis. Several groups have suggested that both brown adipose tissue and lncRNAs could potentially be used as therapeutic targets for the treatment of obesity and obesity-related diseases [24, 40–43]. The prospect of utilizing rs1777538 allele-specific *NFIA-AS2* lncRNAs targeting brown adipose or other obesity-related tissues as obesity therapies is very intriguing and requires additional in-depth studies.

## Supplementary information

Supplementary Figures

Signals associated with BMI identified by meta-analysis of American Indian and GIANT datasets at P < 10^–5^

## Data Availability

The data generated and/or analyzed in the current study is available from the corresponding author upon reasonable request. The summary results of all the variants from our meta-analysis that achieve a *P* < 10^−5^ are available in Supplementary Table 1.
