# Supplementary material for: Identification and characterization of the long non-coding RNA NFIA-AS2 as a novel locus for body mass index in American Indians
Source: Int J Obes (Lond). 2023 Feb 17;47(6):434–42. doi: 10.1038/s41366-023-01278-5 (PMC10212757; doi:10.1038/s41366-023-01278-5)
Supplement: Supplementary file 1 — Supplementary Figures [file 41366_2023_1278_MOESM1_ESM.docx]

**Supplementary Figure 1.**

Flowchart showing the strategy used to identify new BMI loci in American Indians that also have some effect in the GIANT consortium dataset.


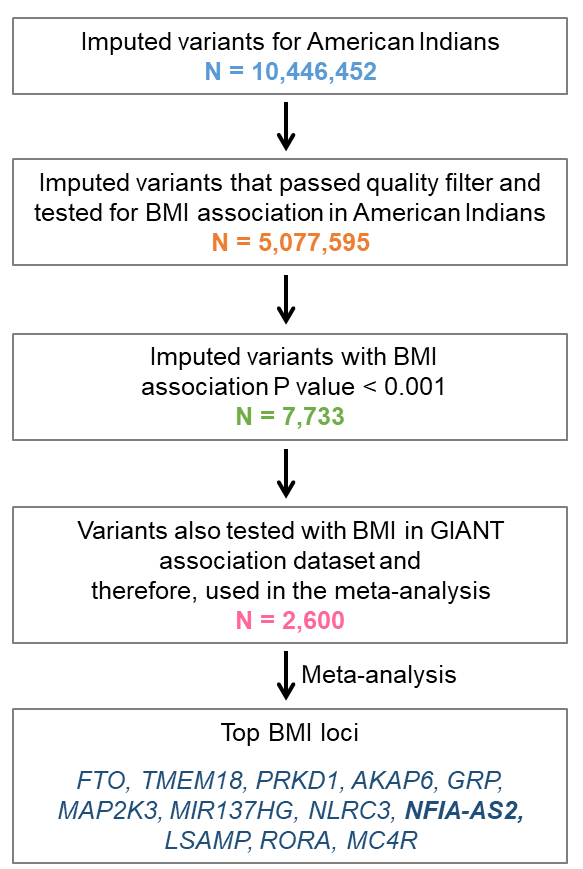


**Supplementary Figure 2.**

Figure showing the variants in the *NFIA-AS2* region significantly associated with BMI in the combined American Indian + GIANT analysis. NFIA-AS2 transcript isoforms V1, V2 and V3 are indicated. The element harboring rs1777538 shows histone marks characteristic of gene enhancers and features chromatin interaction with the promoters of *NFIA-AS2* and long isoform of *NFIA* (ENST00000371191.5)*.*


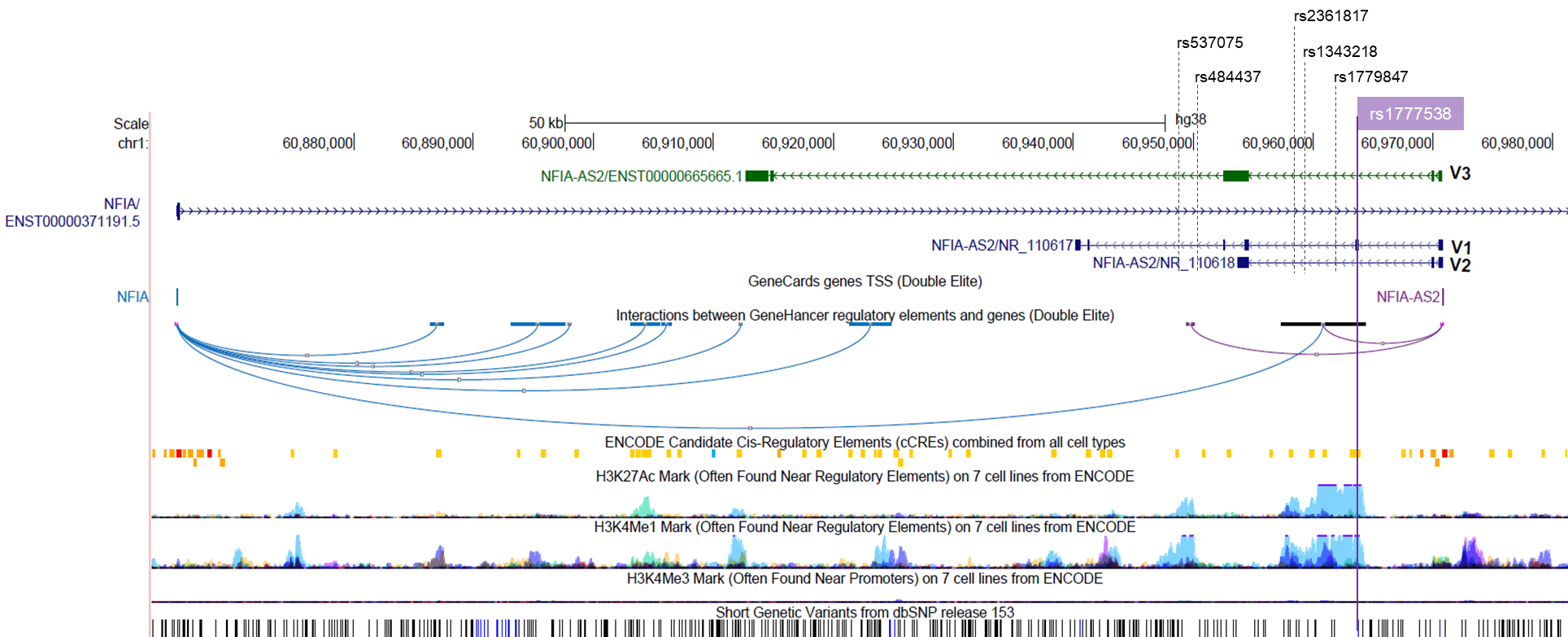


**Supplementary Figure 3.**

Poly(A) RNA sequencing data in brown and white adipose tissue of mouse was deduced from ENCODE v97 and was visualized in UCSC genome browser. *NFIA-AS2* does not exist in mouse.


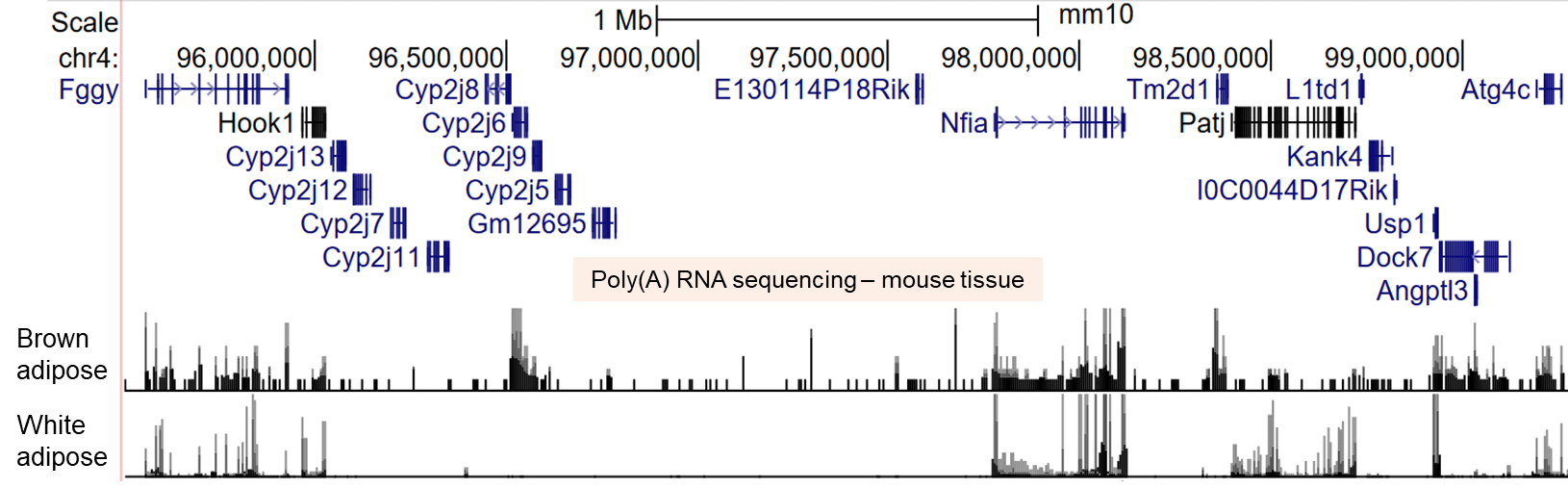


**Supplementary Figure 4.**

Secondary structures for the *NFIA-AS2* transcripts containing either the rs1777538 T or C allele. The rs1777538 T>C variant does not significantly alter RNA secondary structure. Black arrows indicate the rs1777538 T and C nucleotides. The T to C substitution produces only a small change in the H-bonding of the flanking bases. The secondary structures were predicted using SNPfold.

**
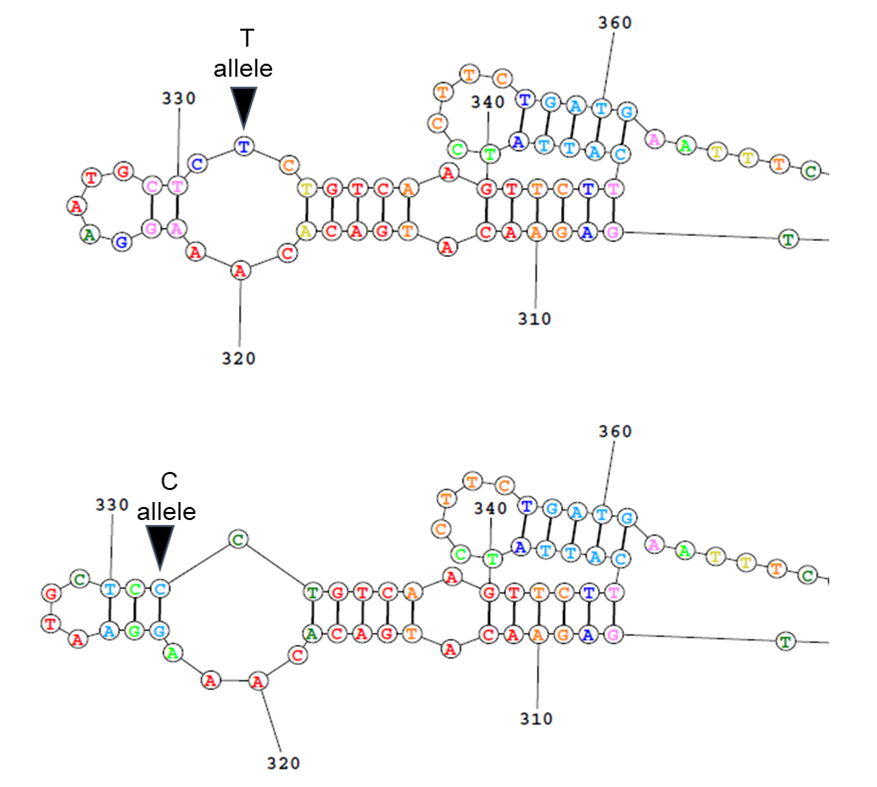
**

**Supplementary Figure 5.**

Expression of neighboring genes of *NFIA-AS2* after treatment with miR6754-5p and miR4270 in HEK293 cells. Expression levels for each gene were normalized with *TBP* and are shown relative to the no miRNA control. Data is presented as mean + SE. The experiments were repeated on three separate days and done in triplicate per experiment.

**
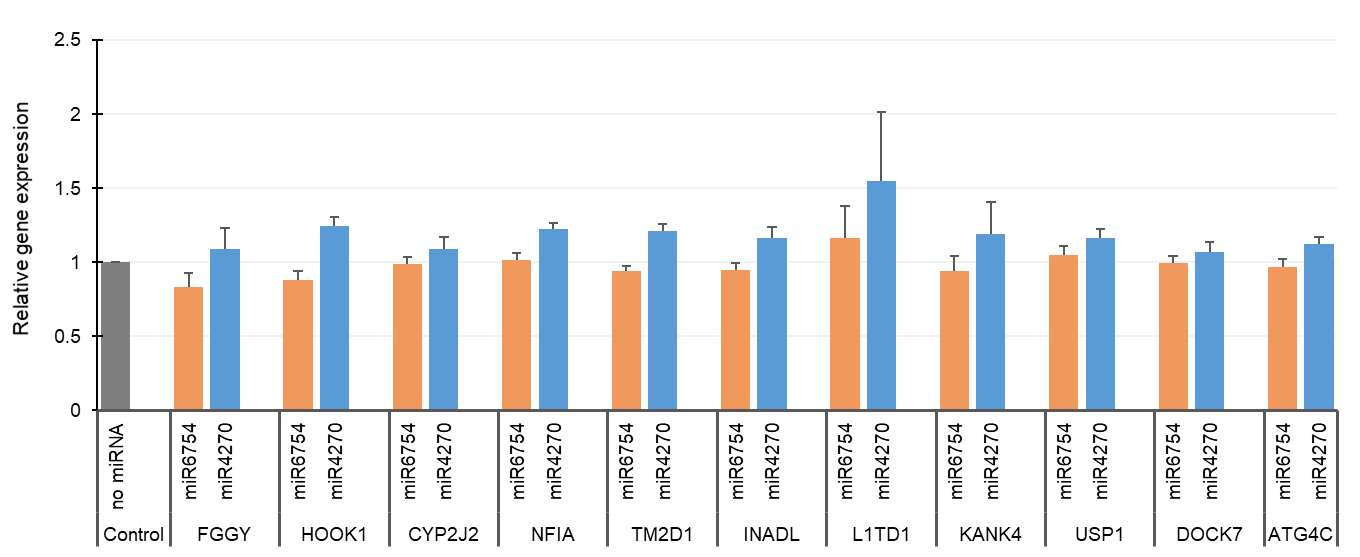
**

**Supplementary Figure 6.**

Expression of different marker genes during differentiation of brown preadipocytes. Expression levels for each gene were normalized with *TBP* and are shown relative to preadipocytes. Data is presented as mean + SE. The experiments were repeated on three separate days and done in triplicate per experiment. Preadipo, preadipocytes.


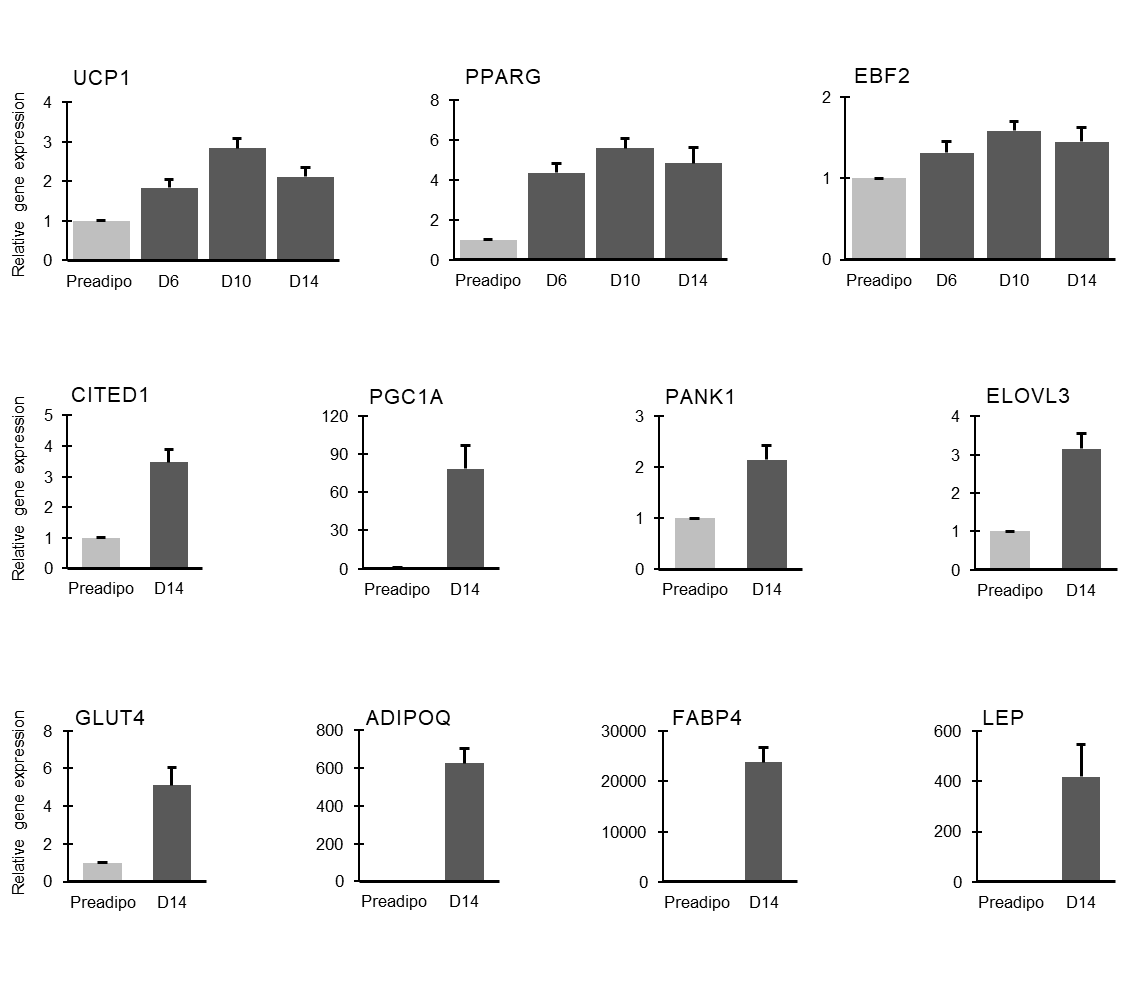


**Supplementary Figure 7.**


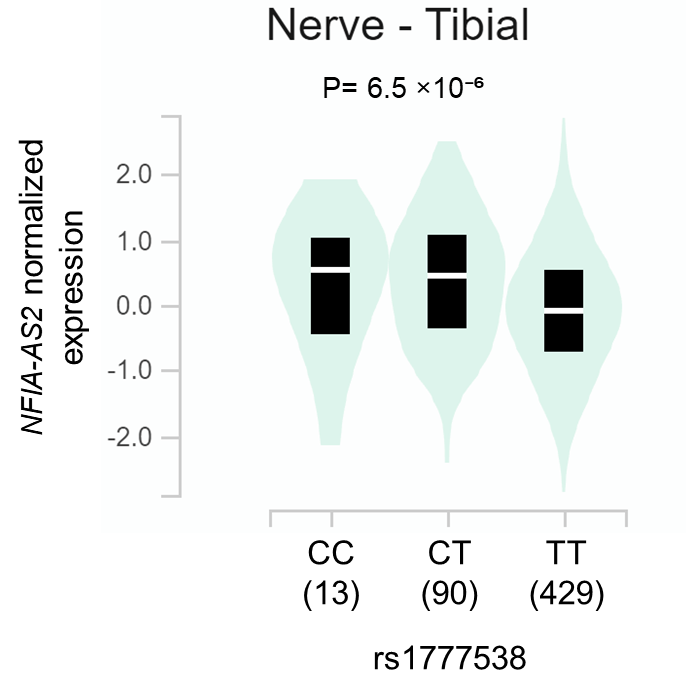
EQTL association data from GTEx database (version 8) showing that TT genotype of rs1777538 is associated with lower *NFIA-AS2* expression in tibial nerve tissue. The violin plot depicts the density of gene expression levels in each genotype and the bar denotes the median values. P = Nominal p value generated for variant-gene pair by testing whether that the slope of a linear regression model between genotype and expression deviates from 0.
